# Non-specific oral and cutaneous manifestations of Coronavirus Disease 2019 in children

**DOI:** 10.4317/medoral.24461

**Published:** 2021-03-27

**Authors:** Elena Bardellini, Maria Pia Bondioni, Francesca Amadori, Federica Veneri, Vassilios Lougaris, Antonella Meini, Alessandro Plebani, Alessandra Majorana

**Affiliations:** 1 Department of Medical and Surgical Specialties, Radiological Sciences and Public Health, School of Paediatric Dentistry-Dental Clinic, University of Brescia, Italy; 2 ASST-Spedali Civili of Brescia, Italy; 3 Department of Medical and Surgical Specialties, Radiological Sciences and Public Health, University of Brescia, Italy; 4 Pediatrics Clinic and Institute for Molecular Medicine A. Nocivelli, Department of Clinical and Experimental Sciences, University of Brescia, Italy; 5 Pediatrics Clinic, ASST-Spedali Civili of Brescia, Italy

## Abstract

**Background:**

Coronavirus Disease 2019 (COVID-19) seems to affect children only marginally, as a result, there is less knowledge of its manifestations in childhood. The purpose of this retrospective cross-sectional study was to investigate the oral and cutaneous manifestations in children affected by COVID-19.

**Material and Methods:**

All the medical records of children with COVID-19 admitted to the Pediatric Clinic- ASST Spedali Civili of Brescia from March to April 2020 were reviewed. The following data were recorded: age, temperature, clinical presentation, oral mucosa lesions, taste alteration and cutaneous lesions.

**Results:**

The medical records of twenty-seven pediatric patients (mean age 4,2 years + 1,7) were analyzed. The clinical presentation of the disease mainly included elevated body temperature and cough. The following oral lesions were recorded: oral pseudomembranous candidiasis (7.4 %), geographic tongue (3.7%), coated tongue (7.4 %) and hyperaemic pharynx (37 %). Taste alteration was reported by 3 patients. Six patients presented cutaneous flat papular lesions.

**Conclusions:**

As for our paediatric sample, COVID-19 resulted to be associated with non-specific oral and cutaneous manifestations.

** Key words:**Child, COVID-19, oral mucosa, taste.

## Introduction

The current outbreak of the novel Severe Acute Respiratory Syndrome Coronavirus-2 (SARS-CoV-2) constitutes a public health emergency of global concern (1-2). This is the third coronavirus to emerge in the human population. It was preceded by the Severe Acute Respiratory Syndrome Coronavirus (SARS-CoV) outbreak in 2002 and the Middle East Respiratory Syndrome Coronavirus (MERS-CoV) outbreak in 2012. Symptoms of coronavirus disease 2019 (COVID-19) in adults include fever, cough and acute respiratory disease, with severe cases leading to pneumonia, kidney failure and even death. Reports on paediatric patients are mainly of Chinese origin and suggest a milder course of COVID-19 in children (3-4). Compared to other respiratory viruses, children seem to have a lower risk of infection since the majority of these illnesses in children were mild or asymptomatic, with few-recorded childhood fatalities attributed to COVID -19 (5). According to Swann, the most common symptoms were fever, cough, nausea/vomiting and shortness of breath. As fever and rhinorrhoea were less common increasing with age, on the contrary nausea and vomiting, abdominal pain, headache and sore throat showed an increasing trend (6).

An interesting outlook during the COVID outbreak was the potential association between Kawasaki disease (KD) and COVID-19. KD can display changes in the lips and in the oral cavity, such as erythema, dryness, fissuring, peeling, cracking, lips prone to bleedings and “strawberry tongue.” As a matter of fact an Italian observational study on paediatric patients by Verdoni *et al*. (7) showed that, during the COVID-19 outbreak, KD had at least a 30 times higher monthly incidence compared to those of the last 5 years in the Bergamo district. Taste alteration was found to be the most reported symptom and thus described oral manifestation during a COVID-19 infection, with a range of prevalence between 5.6% and 92.64% (8-9). Paderno *et al*. (10) reported that in 11% of cases, patients expressed taste alteration as their first symptom of COVID-19 infection, whereas other authors described it as the only symptom detected during the illness itself (11-13). As of now, few studies cite the presence of oral lesions during COVID-19 infection, especially in children. Oral mucosal lesions correlated to COVID-19 have been reported in some Spanish patients, as case series (14-15). In particular, there were a few cases of diffuse oral pain, desquamative gingivitis, ulcers and blisters in adults. Oral manifestations were also reported to be associated with dermatological alterations i.e. erythema multiforme-like eruption. However, no causal association between COVID-19 and cutaneous lesions has been officially demonstrated thus far. Moreover a significant number of cases of chilblains have been observed, mainly in adolescents and young adults with no or mild symptoms compatible with SARS-CoV-2 infection. Therefore a correlation between chilblains and COVID-19 has been suspected, but the pathophysiology of these lesions is still widely debated (16-17).

The purpose of this retrospective study was to investigate the prevalence and characteristics of oral and cutaneous lesions in a group of Italian children affected by COVID-19.

## Material and Methods

This retrospective study was completed in Brescia, one of the Italian cities with the highest number of affected patients. All the medical records of children with COVID-19 admitted to the Pediatric Clinic- ASST Spedali Civili of Brescia from March to April 2020 were reviewed. Inclusion criteria were: (a) pediatric patients (aged 0-14 years old) (b) laboratory evidence of COVID-19 infection (c) signed informed consent.

From each patient’s medical record, the following data were recorded: age, COVID-19 family history, temperature, clinical presentation (cough, rhinorrhoea, breathing difficulty, and saturation), oral mucosa lesions, taste alterations (reported by the child) and cutaneous lesions. This study was approved by the Ethics Committee of ASST-Spedali Civili of Brescia (N. 4055) and followed the Declaration of Helsinki. Written consent was obtained from the caregivers of the patients. The data were inserted on an excel spreadsheet. Descriptive analysis was used for demographic and clinical data.

## Results

A total of 27 children’s medical records were analyzed: patients’ age ranged from 3 months to 14 years of age (mean age 4,2 years + 1,7 years). 17 of the 27 cases (63%) presented a COVID-19 positive family history. Elevated body temperature (>37°C) was the most frequent clinical finding. Most of the patients (15/27, 55.5%) presented a body temperature >38°C while others (10/27 cases, 37%) showed mild febrile conditions (body temperature: 37.5°-38°C). The first reported clinical symptoms included mainly cough (10/27 cases; 37%) and rhinorrhoea (7/27 cases; 25.9%), while difficulty in breathing was rarely registered (5/27 cases; 18.5%), all patients’ oxygen saturation levels constantly remained above 92%. The following oral lesions were recorded: oral pseudomembranous candidiasis (2/27 cases; 7.4%), geographic tongue (1/27 case; 3.7%), coated tongue (2/27 cases; 7.4%) and hyperaemic pharynx (10/27 cases; 37%). Taste alteration was reported by 3 patients (11.1%). Six patients presented cutaneous flat papular lesions (22.2%). The results are summarized in Table 1.

**Table 1:**
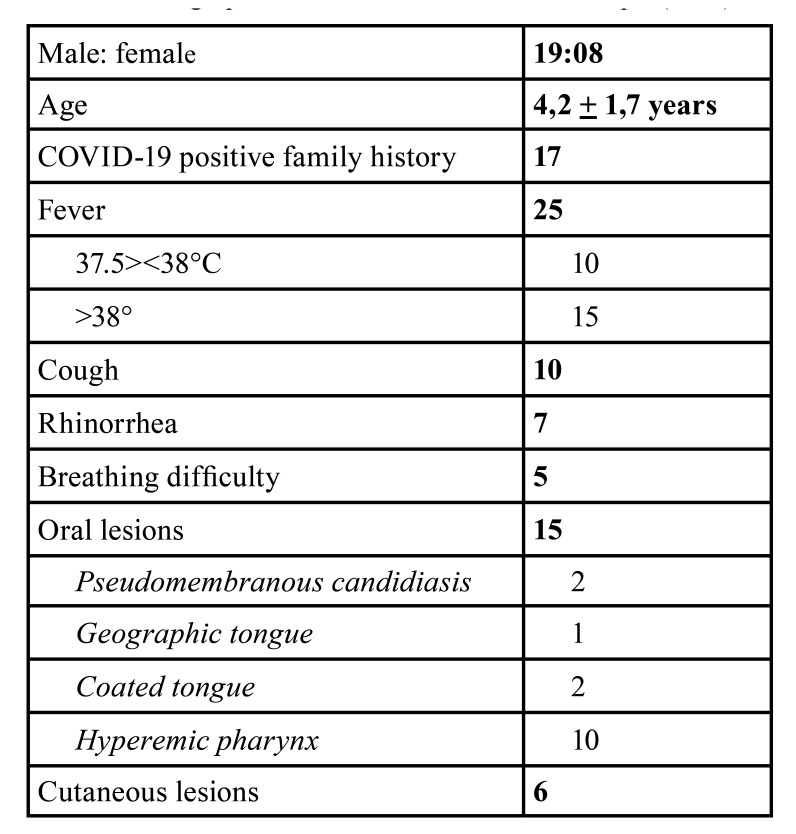
Demographic and clinical features of the sample (n=27).

## Discussion

The clinical presentation of COVID-19 in our paediatric cohort did not differ from that of other studies in paediatric patients (3-4). If compared to adults, the children’s clinical presentations resulted to be overall milder, in terms of cough, breathing difficulty, oxygen saturation, fatigue and abdominal pain, in agreement with Literature (18). A wide spectrum of signs and symptoms were reported in association with novel COVID-19; however, few studies described oral clinical manifestations in patients diagnosed with this disease, none in children as far as we know. Jimenez-Cauhe *et al*. (14) reported observing erythema multiforme (EM)-like oral and cutaneous lesions in 3 hospitalized women (mean age 66.75 years) who showed palatal macules and petechiae. The Authors suggested that the EM-like exanthema might have been another pattern of infection associated with COVID-19 or perhaps a drug-related reaction. Martín Carreras-Presas *et al*. (15) reported 3 cases of vesicle-bullous lesions associated with COVID-19. All cases presented ulcers or blisters in the oral cavity, appearing and advancing during the isolation period. In two of these cases, lesions affected keratinized tissue, as seen in herpes simplex lesions; in the third case, oral lesions affected both keratinized and non-keratinized tissue and were more compatible with EM. However, it must be considered that the available data on oral lesions could have been underreported, reasonably prioritizing the treatment of the respiratory distress first.

In our paediatric cohort, we found the following oral lesions: hyperaemic pharynx, oral pseudomembranous candidiasis, geographic tongue and coated tongue.

The most frequent oral lesion resulted to be hyperaemic pharynx. Acute pharyngitis is an inflammatory syndrome of the pharynx and/or tonsils caused by several different groups of microorganisms. Numerous viruses can cause viral pharyngitis. Pharyngitis can be part of a generalized upper respiratory tract infection or a specific infection localized in the pharynx. Therefore, a mild case of pharyngitis can be considered a finding consistent with the viral origin of COVID-19.

Two patients showed oral pseudomembranous candidiasis, confirmed by a swab of the lesions. In healthy subjects, the percentage of oral candidiasis reported in literature varies from 0.80% to 3.7% in school-age children (19-21). In the present study, candidiasis rates were higher, possibly because newborns and infants were part of our cohort.

Two patients presented a coated tongue. The papillary structure of the tongue dorsum forms a unique ecological site. Its large surface area favours the accumulation of oral debris and microorganisms. Tongue coating is in fact made up of bacteria, large amounts of desquamated epithelial cells released from the oral mucosa, leukocytes from periodontal pockets, blood metabolites and different nutrients. Microscopic research on the tongue's ultrastructure has shown that the formation of tongue coating is associated with the rate of multiplication of epithelial cells. During a viral infection, a coated tongue can be a frequent and reversible occurrence, due to the temporary pH change of the oral cavity. This transient variation can reduce the rate of epithelial desquamation, thus, favouring the accumulation of oral debris and microorganisms (20-22).

One patient had a geographic tongue, which, according to the mother, appeared concurrently with the high fever. The etiopathogenesis of the geographic tongue is still unclear. Several authors have reported a higher prevalence in young children (0-6 years of age) and hypothesized that non-genetic multifactorial factors, including viral infections, could be involved (19-21).

One of the most described oral symptoms in Literature was taste alteration. In most cases of viral upper respiratory infections, the experience of eating food and perceiving flavour can be blunted as a result of rhinitis and nasal obstruction. Whereas during a COVID-19 infection, a loss of smell or taste seems to be a neural process due to viral insult and most commonly results in hyposmia or hypogeusia (24-25). In some cases, anosmia, dysgeusia and hypogeusia appeared to be early presenting symptoms of COVID-19 prior to respiratory symptoms.

However, apart from one single study (25) which used a validated questionnaire, we must take into consideration the fact that most of these documented symptoms were predominantly self-reported, as in our retrospective study. In our sample, 3 children experienced taste alterations and loss of appetite. Nevertheless this data is not reliable, and some symptoms have probably been under-reported, most likely due to the average age of the sample (mainly infants), which obviously prevented them from reporting their symptoms. In addition, another factor worth considering is the impossibility of performing gustatory tests in a retrospective study such as ours.

Cutaneous manifestations in patients with COVID-19 infection are being more and more frequently reported. Several patterns have been described since the initial report by Recalcati (26), including erythematous maculopapular, urticarial, chickenpox-like, purpuric peri-flexural, transient livedo reticularis and acro-ischemic or chilblain-like lesions. In a recent Chinese review, a rash was observed in 0.2% of cases (27). Other studies described dermatologic involvement, including lesions that range from affectation of the hands and feet to vasculitis, rash, urticaria, and varicella-like lesions (28). In our paediatric sample, six children presented cutaneous lesions i.e. non-itchy confluent flat papular lesions of the face and limbs, without any associated lesions in the inner part of the oral cavity. Considering these findings, it is possible that COVID-19 provokes common viral exanthematous lesions, such as those caused by Adenoviruses, Enteroviruses, Influenza A or Para-influenza type 3. Although there are numerous publications about “chilblains”, “pseudo-chilblains”, “chilblain-like lesions”, “covid-toes” or “acral ischemic lesions”, our sample did not show any of these symptoms (29).

Based on our results, we can speculate that there are no specific oral manifestations in children during a COVID-19 infection. It is instead possible to find lesions consistent with those typically found during a common influenza virus infection.
